# Supplementary material for: Metal-organic frameworks as kinetic modulators for branched selectivity in hydroformylation
Source: Nat Commun. 2020 Feb 26;11:1059. doi: 10.1038/s41467-020-14828-6 (PMC7044171; doi:10.1038/s41467-020-14828-6)
Supplement: Supplementary file 1 — Supplementary Information [file 41467_2020_14828_MOESM1_ESM.pdf]

Supplementary Information

Metal-organic frameworks as kinetic modulators for branched selectivity in hydroformylation

Gerald Bauer[1], Daniele Ongari[2], Davide Tiana[3], Patrick Gäumann[1], Thomas Rohrbach[1], Gerard Pareras[3], Mohamed Tarik[4], Berend Smit[2], Marco Ranocchiari*[1]

[1] Laboratory for Catalysis and Sustainable Chemistry, Paul Scherrer Institute, CH-5232 Villigen PSI, Switzerland.

[2] Laboratory of Molecular Simulation, EPFL Valais/Wallis, CH-1951 Sion, Switzerland.

[3] School of Chemistry, University College Cork, College Rd, Cork, Ireland.

[4] Mohamed Tarik, Laboratory for Bioenergy and Catalysis, Paul Scherrer Institute, CH-5232 Villigen PSI, Switzerland.

Correspondence and requests for materials should be addressed to M.R. (email: marco.ranocchiari@psi.ch)

Contents

1. Chemical and Reagents **4**

2. Physical methods **4**

3. Experimental section **5**

3.1. Synthesis 5

MixUMCM-1-PPh$_2$ (29%; [$Zn_4O(BTB)_{4/3}(PPh_2\text{-}BDC)_{0.29}(BDC)_{0.71}]_n$) 5

MOF-74(Zn) 6

MOF-74(Co) 7

MOF-74(Ni) 7

MOF-74(Mg) 7

3.2. Characterization of Metal-Organic Frameworks 8

Powder X-Ray Diffractograms 8

Powder X-Ray Diffractograms before and after catalysis 9

N_2 Adsorption Isotherms (at 77K) 10

Pore Size Distribution 13

3.3. Hydroformylation 15

General procedure for hydroformylation of 1-hexene (Table 1 in the main text and Supplementary Tables 5-9) 15

General procedure for the substrate scope (Table 3 in the main text) 15

Analysis of the conversion and branched to linear ratio 16

Quantification of the oxo-products in Table 3 of the main text 16

Synthesis of $HCo(CO)_3$(MixUMCM-1-PPh$_2$) 17

Hydroformylation with $HCo(CO)_3$(MixUMCM-1-PPh$_2$) 17

Co@MOFs 18

FT-IR of Co@MOFs 18

Hydroformylation with Co@MOF 20

Recycling of Co@MOF 20

Recycling MOF 20

ICP-MS Measurements 21

GC-MS Chromatogram (Table 3 Entry 1 without MOF in Main Text) 23

4. Hydroformylation Catalysis Screening **24**

5. Computational Details **33**

5.1. Interaction Energy Calculations: 33

5.2. Monte Carlo Simulations 36

5.3. Pore Volume Calculations 40

6. DFT Calculations 41

7. Monte Carlo Simulations 43

8. Kinetic Analysis 46

8.1. Rates of formation in homogeneous catalysis 49

8.2. Rates of formation within the pores of UMCM-1-NH_2 50

8.3. Rates of formation within the pores of MOF-74(Zn) 51

9. Supplementary References 52

1. Chemical and Reagents

If not noted differently, all manipulations were carried out under inert atmosphere using standard Schlenk and glove box techniques. The solvents were purified and dried using standard techniques[1] and stored over activated molecular sieves (3 Å). Deuterated solvents were purchased from Armar Chemicals. All other chemicals were purchased from commercial sources. The following chemicals were synthesized according to published procedures: 1,3,5-tri(4-carboxyphenyl)benzene (H_3BTB),[2] 2-diphenylphosphinobenzenedicarboxylic acid (diphenylphosphino-terephthalic acid; Ph_2P-BDC),[3] UMCM-1 $[Zn_4O(BDC)(BTB)_{4/3}]_n$,[4] MixUMCM-1-$NH_2$ (28 mol% NH_2; $[Zn_4O(BDC)_{0.72}(NH_2\text{-}BDC)_{0.28}(BTB)_{4/3}]_n$),[4] UMCM-1-$NH_2$ ($[Zn_4O(NH_2\text{-}BDC)_1(BTB)_{4/3}]_n$),[4] MIL-101(Al),[5] MIL-101(Cr).[6]

2. Physical methods

NMR spectra were recorded on a Bruker Avance 500 ^1H NMR chemical shifts were referenced to residual solvent peak as determined relative to TMS (δ = 0 ppm). ^{31}P spectra were referenced to $NH_4H_2PO_4$ (solid state; δ = 1.0 ppm) and 85% H_3PO_4 in D_2O (solution NMR; δ = 0.0 ppm). GC measurements were conducted on an Agilent 6890 GC equipped with an HP-5 column and FI-detector. GC-MS measurements were conducted on an Agilent 7890A GC equipped with HP-5 column and an Agilent 5975C XL MSD. Powder-XRD measurements were performed at room temperature on a Bruker AXS D8 Advance Bragg-Brentano Diffractometer equipped with a Braun detector at 40 kV, 40 mA with CuKα (λ = 1.54 Å) radiation, step size 0.02 s and a 2θ range of 4-40°. The nitrogen adsorption isotherms are calculated by the BET (Brunauer-Emmet-Teller) method and the measurements were performed by the Micromeritics Tristar II 3020 equipped with a VacPrep 061 degassing station. The pore size distribution was

calculated using the Horvath-Kawazoe model modified by Saito and Foley for cylindiral pores. UPLC experiments were performed on a Waters Acquity UPLC H-Class system equipped with a UV/Vis detector, a Waters BEH C18 (1.7 µm) column and Acquity QDa mass detector. For SEM images, thin layers of MOF-74(Zn) were spread on a flat surface of carbon tape on top of an aluminium stump. The sample was coated with a 5 nm thick carbon layer using a CCU-010 Carbon Coater Safematic. The images were collected using a SEM JSM-7100F JEOL scanning electron microscope at 15.0 keV with a working distance of 11 mm. Optical microscope images were acquired using a Leica M165 C microscope equipped with a Leica DFC425 C camera. The images were processed with Leica Application Suite 4.12.0. FT-IR spectra were measured on a ThermoFischer Nicolet iS50 FT-IR using its ATR cell.

3. Experimental section

3.1. Synthesis

MixUMCM-1-PPh$_2$ (29%; [$Zn_4O(BTB)_{4/3}(PPh_2\text{-}BDC)_{0.29}(BDC)_{0.71}]_n$)

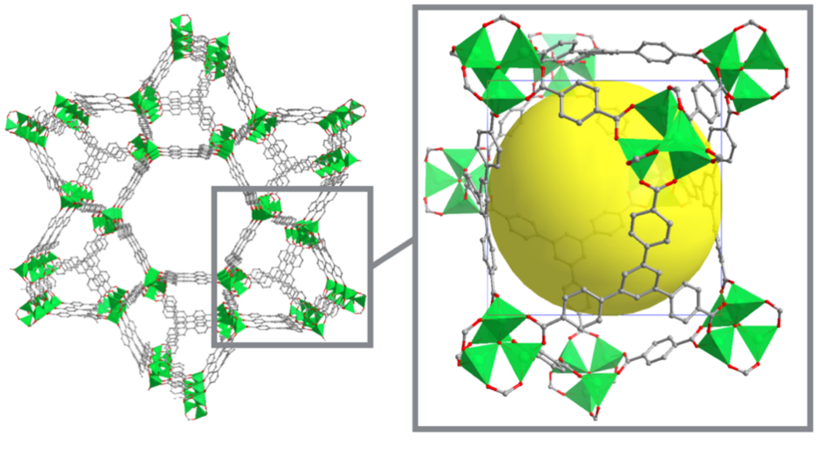

$[Zn_4O(btb)_{4/3}(bdc)_{0.71}(PPh_2\text{-}bdc)_{0.29}]_n$
MixUMCM-1-PPh$_2$ (29%)

Supplementary Figure 1 Structure of MixUMCM-1-PPh$_2$ (29%).

Based on literature procedure:[4]

$Zn(NO_3)_2{\bullet}6H_2O$ (1.61 g, 5.41 mmol), terephthalic acid (BDC; 150 mg, 0.90 mmol), diphenylphosphino-terephthalic acid (PPh_2-BDC; 178 mg, 0.51 mmol) and H_3BTB (212 mg, 0.48 mmol) were dissolved in DMF (50 mL). The solution was divided into 10 mL portions and transferred into 20 mL glass vials. The vials were placed in a sand bath and transferred into an isothermal oven heated at 85 °C for 72 h while continuously flushing with N_2.

After cooling down the oven to room temperature, the vials were removed from the oven and the mother liquor was decanted. The crystals were collected and washed with fresh DMF (3 × 15 mL) and soaked in $CHCl_3$ (15 mL) for 3 days with the replacement of the fresh $CHCl_3$ each 24 h. The obtained crystals were stored in toluene until use.

<u>PPh_2-BDC loading</u>

The MOF sample (3 mg dry mass) was placed in a NMR tube and suspended in DCl solution (0.1 mL; 20% in D_2O) using an ultrasonification bath. The solids were then dissolved by adding DMSO-d_6 (0.5 mL) and analyzed by NMR.

MOF-74(Zn)

In a 20 ml microwave tube, 2,5-dihydroxyterephthalic acid (200 mg; 1.01 mmol) and $Zn(acac)_2{\bullet}H_2O$ (568 mg; 2.02 mmol) were dissolved in DMF (19 ml) and H_2O (1 ml) to give a yellow solution. The reaction mixture was stirred at 130°C for 60 min in a microwave oven. The solid of the reaction mixture was filtered by membrane filter, washed with DMF, H_2O and EtOH and dried in the vacuum oven. Yield: 388 mg (81% calculated on dry MOF).

MOF-74(Co)

In a 20 ml microwave tube, 2,5-dihydroxyterephthalic acid (400 mg, 2.02 mmol) and $Co(NO_3)_2•6H_2O$ (1.82g, 6.26 mmol) were dissolved in DMF/EtOH/H_2O (20 mL, Ratio = 1/1/1) to give a yellow solution. The reaction mixture was stirred at 130°C for 1.5 h in a Biotage Initiator+ microwave oven. Yield: 850 mg (40% calculated on dry MOF). The resulting solid was filtered by membrane filter, washed with DMF, EtOH and *tert*-butylmethylether and dried *in vacuo*.

MOF-74(Ni)

Based on a literature procedure:[7]

In a 250 ml round-bottomed flask, 2,5-dihydroxyterephthalic acid (2.0 g, 10.09 mmol) and $Ni(NO_3)_2•6H_2O$ (3.1 g, 31.29 mmol) were dissolved in DMF/EtOH/H_2O (100 mL, Ratio = 1/1/1) to give a brown solution. The solution was equally distributed into five 20 mL microwave tubes. The reaction mixture was stirred at 130°C for 1.5 h in a microwave oven giving a brown suspension. The resulting solid was filtered by membrane filter, washed with DMF, EtOH and *tert*-butylmethylether and dried *in vacuo*. Yield: 3.75 g (53% calculated on dry MOF).

MOF-74(Mg)

In a 2000 mL round bottom flask, 2,5-dihydroxyterephthalic acid (2.58 g, 13.00 mmol) and $Mg(NO_3)_2•6H_2O$ (6.67g, 26.00 mmol) were dissolved in DMF (1240 ml) and H_2O (60 ml) to give a yellow solution. The reaction mixture was stirred at reflux (bath 140°C) for 22 h. The resulting solid was filtered by membrane filter, washed with DMF, EtOH and *tert*-butylmethylether and dried *in vacuo*. Yield: 3.15 g (57% calculated on wet MOF).

3.2. Characterization of Metal-Organic Frameworks

Powder X-Ray Diffractograms

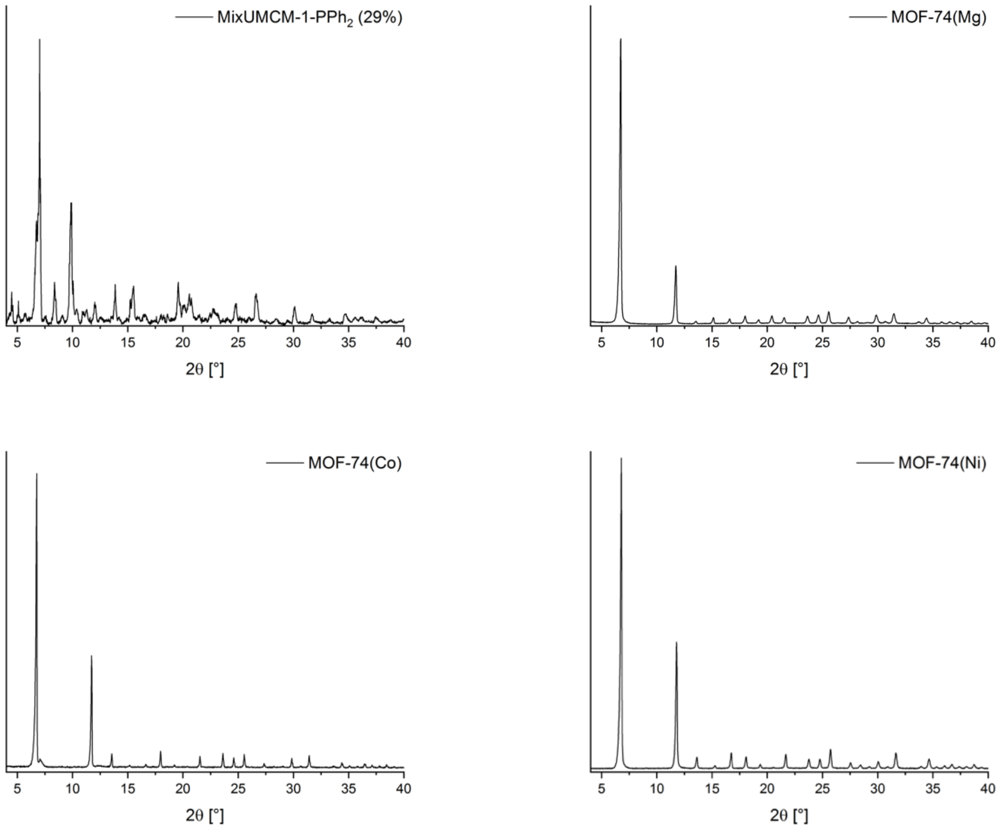

Supplementary Figure 2 Powder X-Ray pattern of the MOFs used in this work. MixUMCM-1-PPh_2 (29%), MOF-74(Mg), MOF-74(Ni) and MOF-74(Co) when starting from top left and going clockwise.

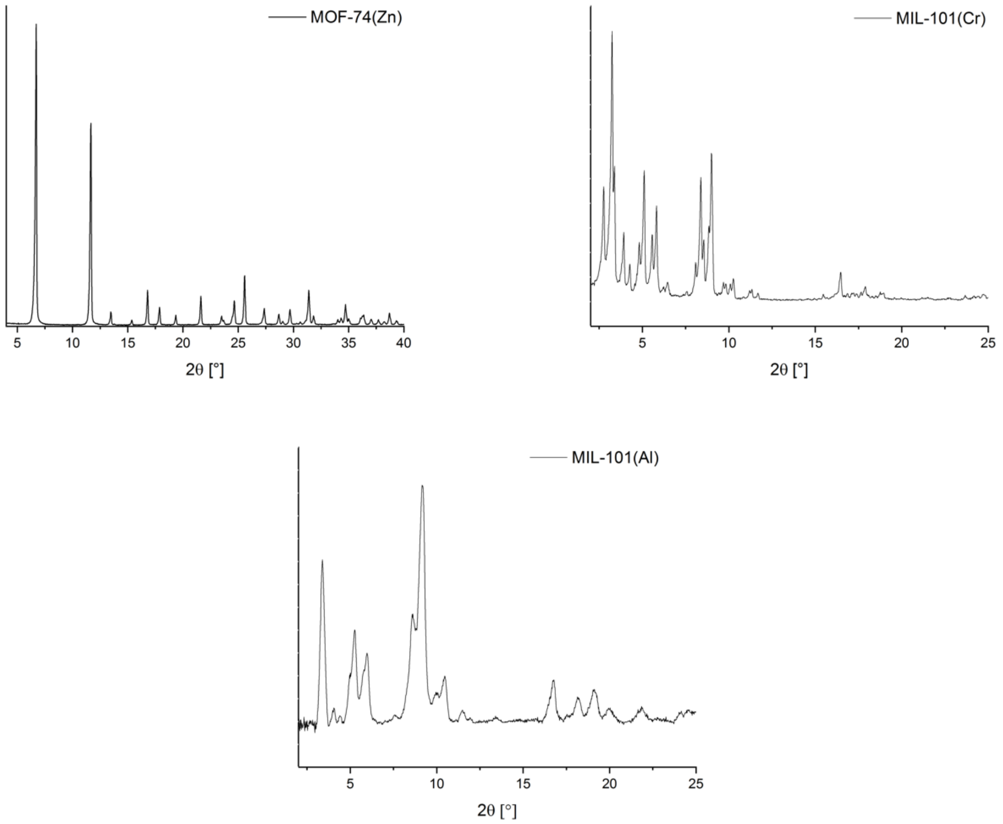

Supplementary Figure 3 Powder X-Ray pattern of the MOFs used in this work. MOF-74(Zn), MIL-101(Cr) and MIL-101(Al) when when starting from top left and going clockwise.

Powder X-Ray Diffractograms before and after catalysis

MixUMCM-1-NH$_2$ (28%) **MOF-74(Zn)**

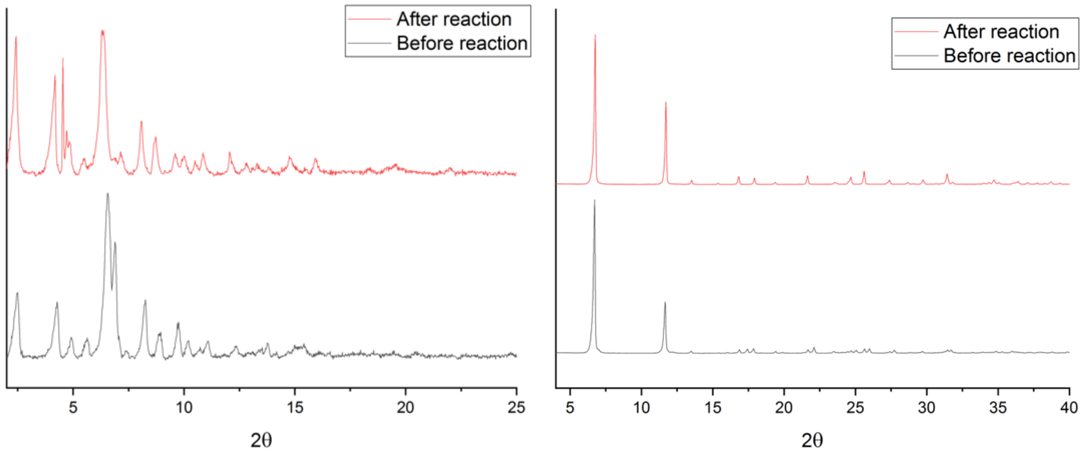

Supplementary Figure 4 Powder X-Ray pattern of MixUMCM-1-NH$_2$ (28%) (left) and MOF-74(Zn) (right) after (red) and before (black) catalysis respectively.

N_2 Adsorption Isotherms (at 77K)

UMCM-1 samples preparation:

Materials with UMCM-1 topology were measured after work up. The supernatant solution was decanted off and the MOF was dried under a stream of Ar. The sample was then transferred to a BET sample vial and activated at 120 mTorr and 120°C. The weight was determined after activation. For BET measurements after catalysis, the reaction mixture was filtered and the solid was washed with $CHCl_3$ (3 x 10 ml) before it was activated and measured as stated above.

MOF-74(M) samples preparation:

The MOF-74(M) materials were transferred to a BET sample vial and activated at 120 mTorr and 250°C. The weight was determined after activation. For BET measurements after catalysis, the reaction mixture was filtered and the solid was washed with THF and EtOH (both 3 x 10 ml) followed by Soxhlet extraction (THF, 3 d). The obtained solid was activated and measured as stated above.

BET Numbers:

Supplementary Table 1 BET numbers of the MOFs used in this work.

Entry	MOF	BET Number ($m^2 \cdot g^{-1}$)	BET Number after catalysis ($m^2 \cdot g^{-1}$)
1	MixUMCM-1-PPh_2 (29%)	1470	n/a
2	MixUMCM-1-NH_2 (28%)[a]	1760	600 (After Table 3 Entry 1)
3	MixUMCM-1-NH_2 (28%)[b]	2870	2980 (After Table 1 Entry 4)
4	MOF-74(Mg)	1370	n/a
5	MOF-74(Co)	1260	n/a
6	MOF-74(Ni)	1360	n/a
7	MOF-74(Zn)	990	20 (After Table 3 Entry 1)
8	MOF-74(Zn)	1000	150 (After Table 1 Entry 6)
9	MIL-101(Al)	2790	n/a
10	MIL-101(Cr)	2810	n/a

a. Sample used in the experiments of Table 1 Entries 3 and 5 in the manuscript. b. Sample used in Table 1 entry 5, Supplementary Tables 7-9, for pore size analysis, for SEM analysis, and for impregnation experiments (Supplementary Table S2).

Nitrogen Physisorption before and after catalysis (Table 1 Entry 3 (A), Table 1 Entry 8 (B))

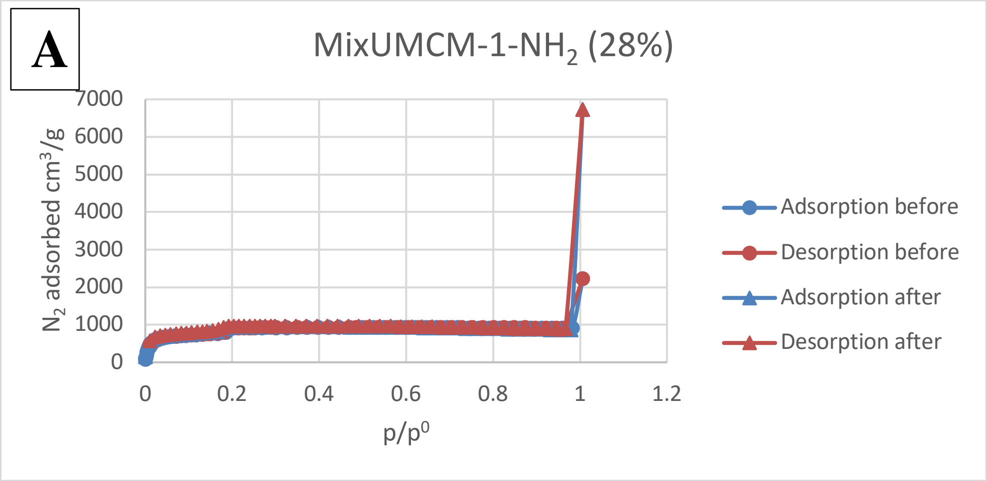

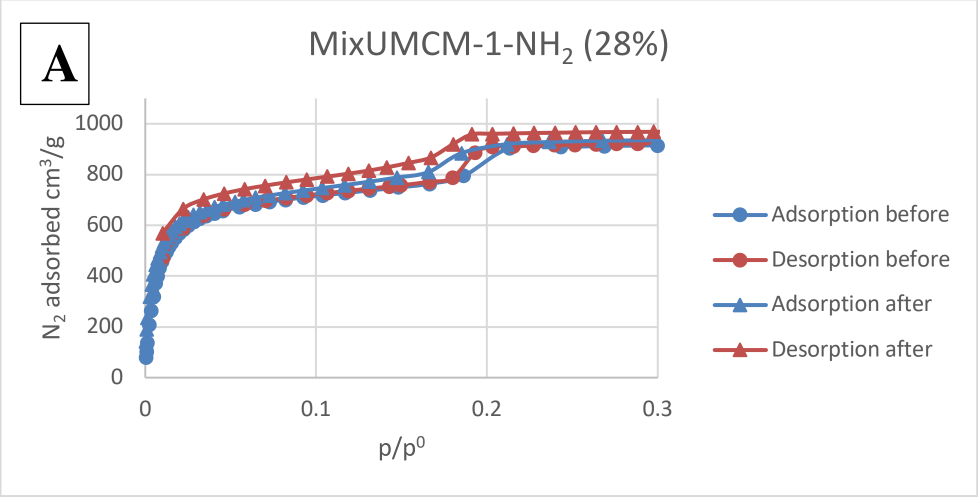

Supplementary Figure 5 Nitrogen Physisprtion curves of MixUMCM-1-NH$_2$ (28%). Blue and red symbols representing the adsorption and desorption branch respectively. Circles show the values before and triangles the ones after catalysis.

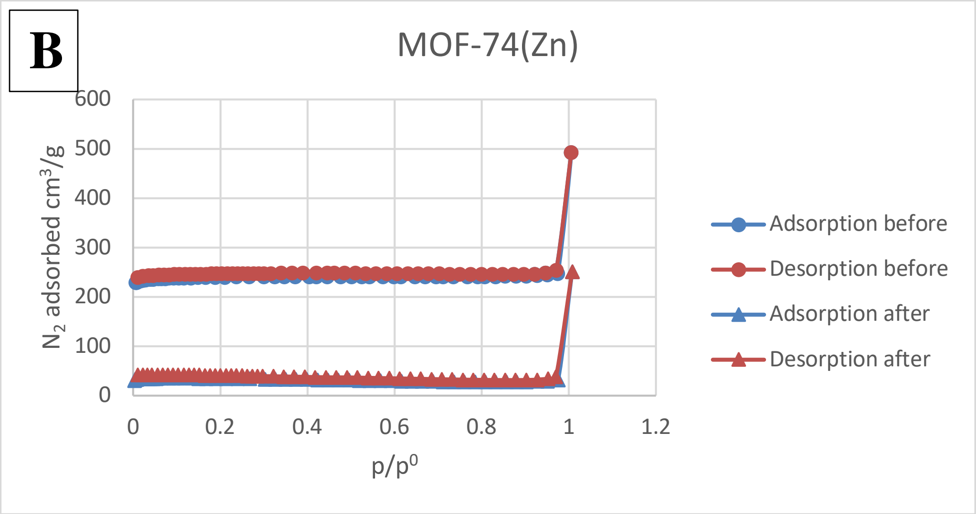

Supplementary Figure 6 Nitrogen Physisorption curves of MOF-74(Zn). Blue and red symbols representing the adsorption and desorption branch respectively. Circles show the values before and triangles the ones after catalysis.

Pore Size Distribution

Below are the Horvath-Kawazoe differential pore volume plots of the samples before and after catalysis under conditions of Table 1 in the main text. The mesopores in MixUMCM-1-NH_2 (28%) are not detected because the model is optimized for micropores.

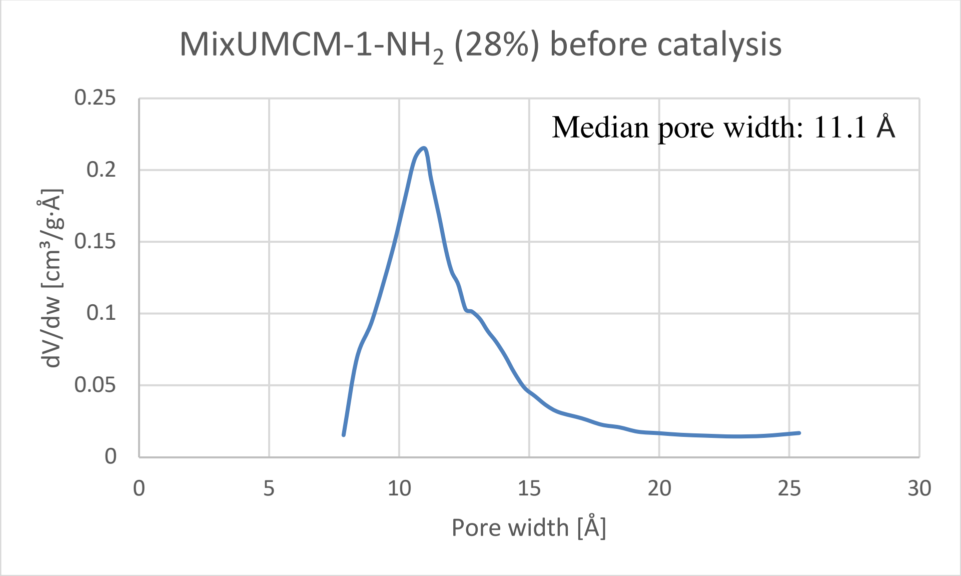

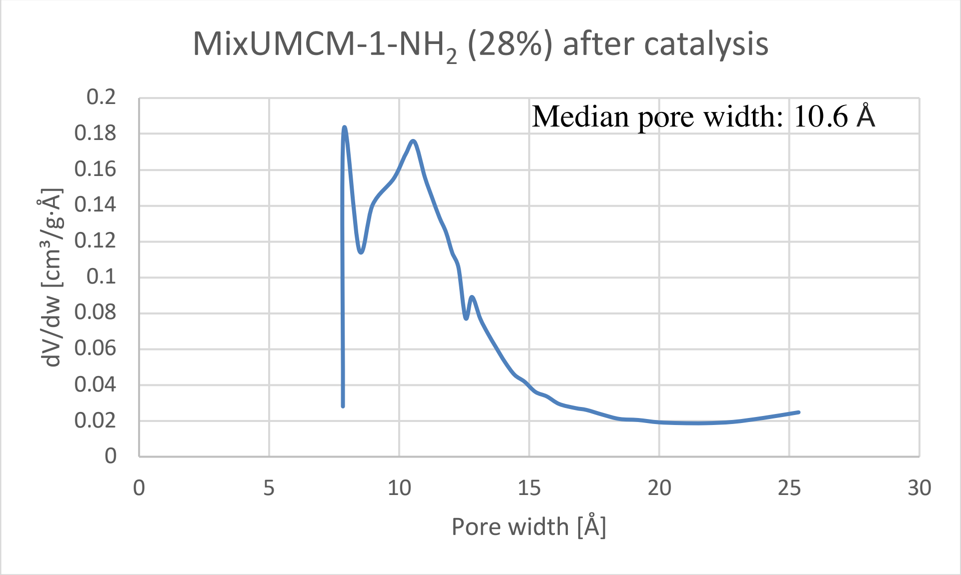

Supplementary Figure 7 Pore size distribution of MixUMCM-1-NH_2 (28%). Calculated by Horvath-Kawwazoe model for MixUMCM-1-NH_2 (28%) before (top) and after (bottom) catalysis.

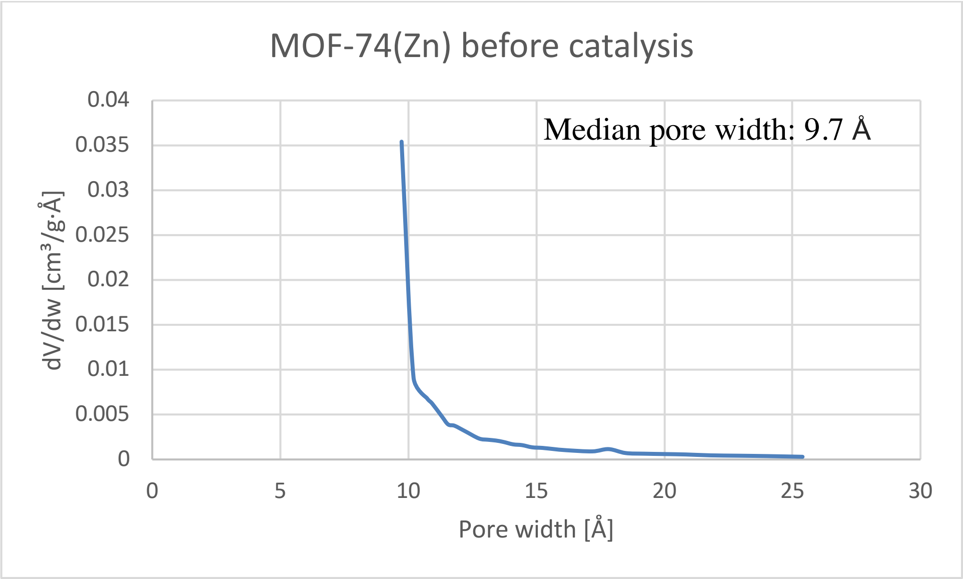

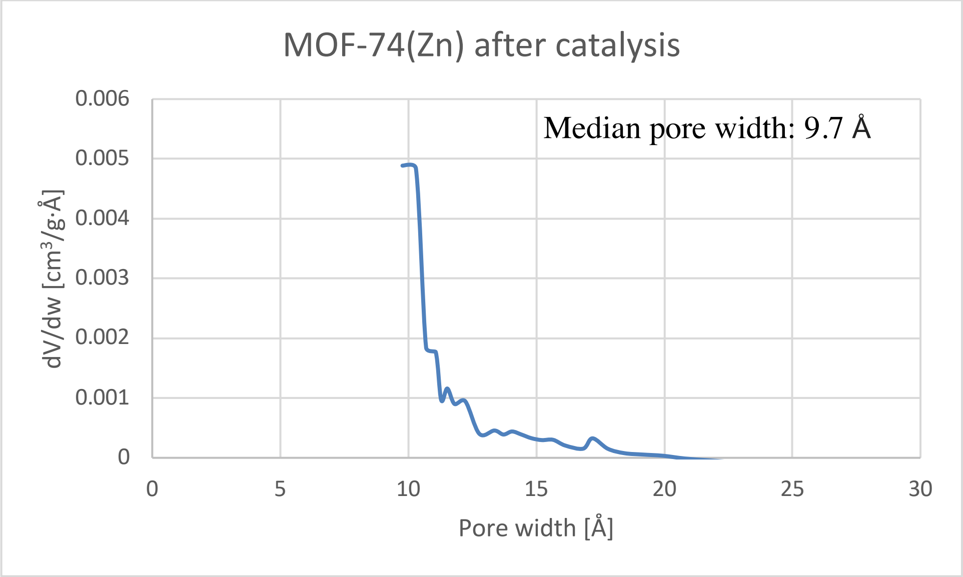

Supplementary Figure 8 Pore size distribution of MOF-74(Zn). Calculated by Horvath-Kawwazoe model for MOF-74(Zn) before (top) and after (bottom) catalysis.

3.3. Hydroformylation

General procedure for hydroformylation of 1-hexene (Table 1 in the main text and Supplementary Tables 5-9)

A stock solution of $Co_2(CO)_8$ in 1-hexene was prepared inside the glove box. In a 1.5 mL GC crimp vial the MOF was weight in and activated at 150 °C overnight. The MOF was then suspended in 250 µL Co/hexene stock solution and the vial was closed. The vial put in a 50 mL Premex® autoclave and purged with Ar. The autoclave was then briefly opened (under a flow of Ar) and the septum was pierced with a needle. The autoclave was closed, then syngas ($CO:H_2$ = 1) was introduced and the reaction mixture was heated to 100°C for 18 hours.

The reaction mixture was cooled to room temperature and the pressure was slowly released. The samples were topped up with 1 mL acetonitrile and 50 µL were transferred in a GC vial filled with acetonitrile and *p*-cymene. The conversion and branched:linear ratios were obtined by GC-FID with *p*-cymene as external standard using the same response factor (R_f) for all aldehyde products. In addition to the hydroformylation products, the chromatograms showed traces of unknown compounds, which never exceeded 5% of the total area of 1-hexene and hydroformylation products.

General procedure for the substrate scope (Table 3 in the main text)

The MOF (10 mol% to the olefin for MOF-74(Zn), 1 mol% to the olefin for UMCM-1 derivatives) was placed in a crimp vial, which was closed with a crimp cap and pierced with a needle. In the case of MOF-74(Zn), the vial was placed into a round-bottom flask and activated at 150 °C in vacuum for 24 h. The round-bottom flask was allowed to cool to room temperature but remained under vacuum until it was introduced into a nitrogen-filled glovebox. UMCM-1 derivatives were stored in the glovebox and weighed in without pervious activation. $Co_2(CO)_8$ (1.5 mol%) was dissolved in the olefin (500 µL), and the whole solution was added to the MOF.

The vials were placed into a 50 mL Premex® autoclave and purged with Ar several times. The valves to and from the autoclave were closed and the syngas line flushed once. Syngas pressure ($CO:H_2$ 1:1, 30 bar) was applied and the autoclaves heated at 100 °C for 17 h. The autoclave was allowed to cool down to room temperature before the pressure was released slowly over 15 min. The autoclave was flushed with nitrogen before it was opened to remove additional syngas.

Analysis of the conversion and branched to linear ratio

The content of the reaction vials was transferred into a 5 ml volumetric flask and filled with THF. The MOF was extracted for 30 min before 200 µl of the suspension was added to 800 µl of a solution of the internal standard (p-cymene 0.048 M in THF). This suspension was filtered and analyzed by GC-FID. The branched to linear ratio was calculated from the ratio between the integrals of the isomers assuming the same R_f. Conversion and aldehyde yield in Table 3 and Supplementary Table 10 were calculated with GC-FID upon calibration of the olefins and the linear aldehydes with p-cymene. The R_f of all olefin isomers were assumed the same. The R_f of all aldehydes were assumed the same. The total oxo products yield was determined as described below.

Quantification of the oxo-products in Table 3 of the main text

The total yield of oxo products (aldehydes + aldol products), which is an indication of the performance of hydroformylation, was calculated since we always observed aldol products at the high conversion in Table 3 (See GC-MS data above). Desorbing the aldehydes from the pores of the MOFs without decomposing them after catalysis was a challenge.

The method to yield determination of the oxo products was:

1) measure the mass of the raw product after catalysis;

2) calculate the mass of the pure oxo products by removing the mass of non converted olefin and of impurities detected by GC;

3) calculate mol of oxo products with the corrected mass and the molar mass of the corresponding aldehydes. This is a safe assumption since aldol products have molar mass multiple to that of the aldehyde.

Synthesis of $HCo(CO)_3$(MixUMCM-1-PPh_2)

Inside the glove box, $Co_2(CO)_8$ (18 mg, 108 µmol) and MixUMCM-1-PPh_2 (29 mol%) (92 mg, 25.2 µmol PPh_2) were weight into a 10 mL crimp vial and toluene (5.0 mL) was added. The vial was placed in a 50 mL Premex® autoclave and purged with Ar. The autoclave was then briefly opened (under a flow of Ar) and the septum was pierced with a needle. The autoclave was closed, then syngas (10 bar, $CO:H_2 = 1$) was introduced and the reaction mixture was kept at room temperature for 72 hours. After pressure release the sample was immediately placed inside the glove box and the supernatant reaction solution was decanted off. The residual MOF was quickly washed with 2 x 0.5 mL toluene and left open to the atmosphere to evaporate the excess solvent and submerged in toluene (5 mL) for 24 hours in order to leach the excess Co-species. This procedure was repeated twice.

Hydroformylation with $HCo(CO)_3$(MixUMCM-1-PPh_2)

Inside the glove box, a 1.5 mL GC crimp vial was charged with pre-treated MOF (4.5 mg dry mass). The MOF was then suspended in 250 µL neat 1-hexene. The vial was then closed and put in a 50 mL Premex® autoclave and purged with Ar. The autoclave was then briefly opened (under a flow of Ar) and the septum was pierced with a needle. The autoclave was closed again, the syngas ($CO:H_2 = 1/1$) was introduced and the reaction mixture was heated to 100°C for 17 hours.

Co@MOFs

MixUMCM-1-NH_2 (28 %) was activated at 100 °C under vacuum for 24 h. MOF-74(Zn) was activated in a round-bottom flask at 200 °C under vacuum for 8 h. The MOF was transferred to a nitrogen-filled glove box. A solution of $Co_2(CO)_8$ (99.2 mg) in DCM (1 ml) was prepared, diluted with DCM (1.0 x, 2.0 x and 3.0 x, See Supplementary Table 2). The resulting solutions were slowly added to the MOFs via a syringe. To make the mixture as homogeneous as possible, MOFs were stirred carefully with a spatula. The samples were allowed to dry for at least 8 h at room temperature in the glovebox before they were used for catalysis.

Supplementary Table 2 Overview incipient-wetness impregnated samples.

Entry	Sample	Conc. Solution [M]	Amount Solution [µl]	Amount MOF [mg]	Co loading Wt% $Co_2(CO)_8$
1	Co@MixUMCM-1-NH_2-13	0.293	300	196.5	13
2	Co@MOF-74(Zn)-9.3	0.290	200	191.8	9.3
3	Co@MOF-74(Zn)-4.3	0.145	200	220.5	4.3
4	Co@MOF-74(Zn)-3.8	0.097	200	164.1	3.8

FT-IR of Co@MOFs

Fourier transform infrared spectroscopy (FT-IR) of UMCM-1-NH_2 impregnated with $Co_2(CO)_8$ showed CO stretches between 1800 cm^{-1} and 2000 cm^{-1} similar to those of the neat pre-catalyst $Co_2(CO)_8$. This indicates that the structure of the pre-catalyst is similar in the presence of the MOF or without it. FT-IR of MOF-74 impregnated with $Co_2(CO)_8$ showed no CO stretches between 1800 cm^{-1} and 2000 cm^{-1} with contomitant colour change from dark black to light grey indicating fast decomposition of $Co_2(CO)_8$ under O_2 and/or moisture. The IR spectra and comparison with the $Co_2(CO)_8$ and the MOFs are shown below.

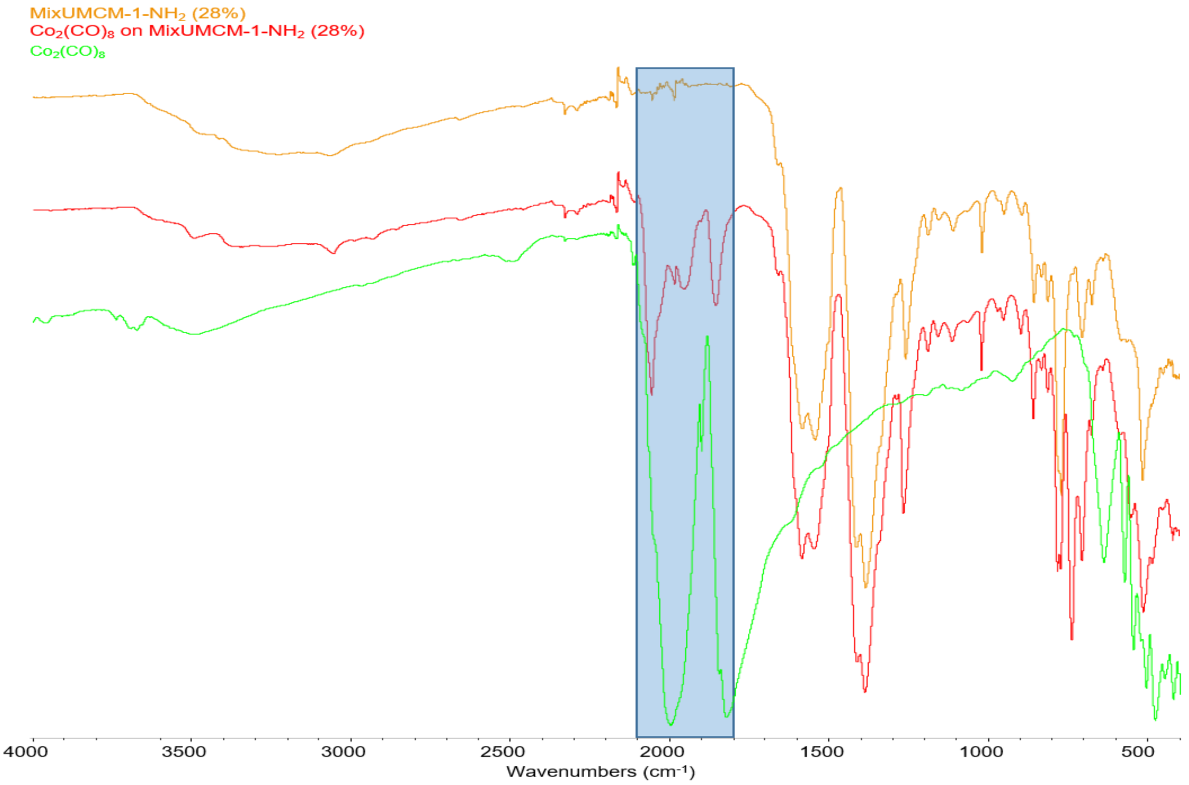

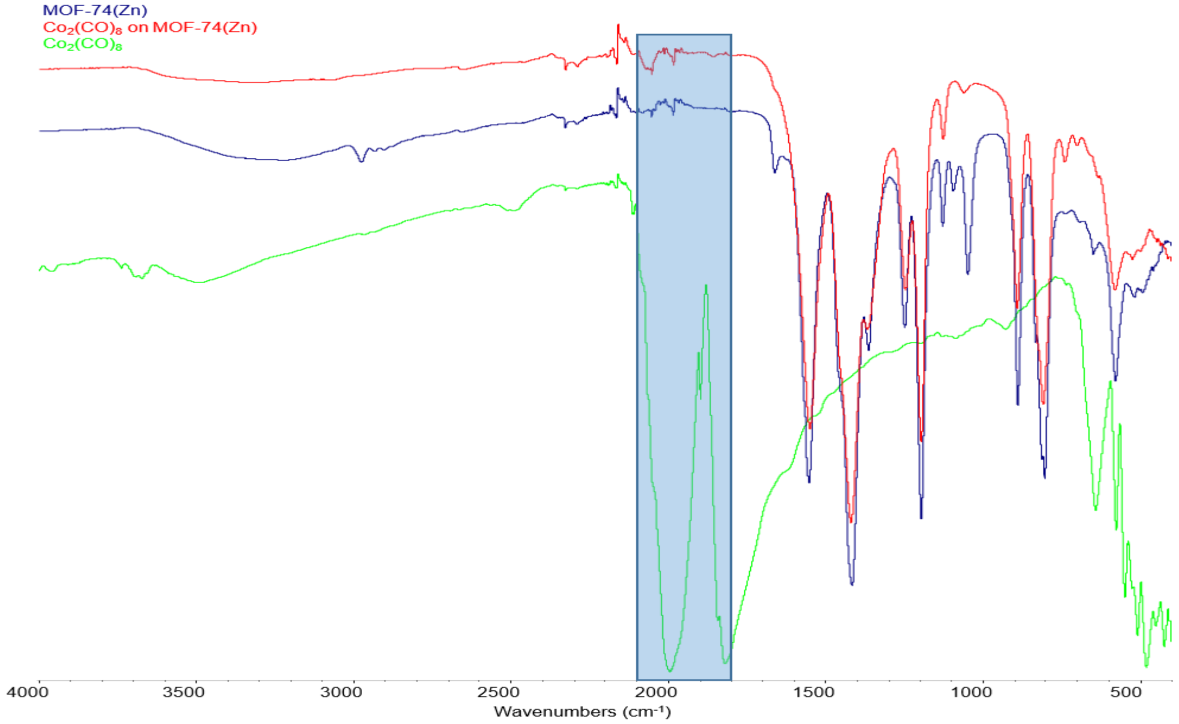

Supplementary Figure 9 FT-IR spectra. Pristine MOF (top), catalyst-loaded MOF (middle) and catalyst (bottom) for MixUMCM-1-NH$_2$ (28%) (top spectrum) and MOF-74(Zn) (bottom spectrum).

Hydroformylation with Co@MOF

Co@MOF (amount in Supplementary Table 11) was added to a 2 ml crimp vial in a nitrogen-filled glove box. 1-Hexene (500 µl, 4.0 mmol, 1.0 eq.) was added to all vials before they were closed with a crimp cap and taken out of the glove box. The vials were placed in the autoclave which was flushed several times before syngas pressure was set to 30 bar at room temperature. The reactor was heated to 100°C leading to a pressure of 35 bar and the substrates were allowed to react for 16 h. The autoclave was cooled down to room temperature and the remaining syngas pressure was slowly released to avoid spilling.

Recycling of Co@MOF

After the first catalytic run, the reaction mixture was removed with a syringe. The MOF was washed once by 1-hexene (1 mL) and then the solvent extracted with a syringe. Fresh 1-hexene (500 µl, 4.0 mmol) was added again, the vials were closed and the reaction was carried out as stated above.

Recycling MOF

After the first catalytic run, the MOF was filtered off and washed with $CHCl_3$ (3 x 10 ml) in the case of MixUMCM-1-NH_2 (28%). MOF-74(Zn) samples were washed with THF (3 x 10 ml) and EtOH (3 x 10 ml) before they were purified by Soxhlet extraction (5 d, THF). The purified MOFs were used for hydroformylation following the standard procedure of Table 1 in the main text.

ICP-MS Measurements

0.5 mL H_2O_2 (30%), 1 mL H_2SO_4 (96%) and 1 mL HCl (30%) were added to about 20 mg from each MOF samples (with and without Co) and then digested by using a high-pressure microwave unit (Anton Paar). Afterwards the digested samples were diluted with MilliQ H_2O to a total volume of 50 mL. Another two dilution steps were carried out with a 1% HCl solution resulting in total dilution factor of ~ 8.0 105.

The hexene liquid solutions were heated for several hours (at about 60°C) and then 1 mL HCl (30%) was added to each sample. The obtained solutions were then diluted in 2 steps by a factor of 2 104.

4 standard solutions of Co, P and Zn were prepared with concentration in the range of 0 to 100 ppb (ng/mL). The analysis was performed on an ICP-MS 7700x after optimizing the system for high sensitivity and low oxide rate. The isotopes ^{59}Co, ^{31}P and ^{66}Zn were measured and the corresponding three elements in all the samples were quantified by using external calibration procedure.

Supplementary Table 3 Co-Loading in MixUMCM-1-NH_2 (28%) after catalysis dependent on the pressure.

Entry	Syngas pressure (bar)	Co-uptake (%)
1	19	56
2	30	73
3	55	82
4	72	85
5	94	67

Supplementary Table 4 Co-Loading in MOF-74(Zn) after catalysis dependent on the pressure.

Entry	Syngas pressure (bar)	Co-uptake (%)
1	30	60
2	61	36

GC-MS Chromatogram (Table 3 Entry 1 without MOF in Main Text)

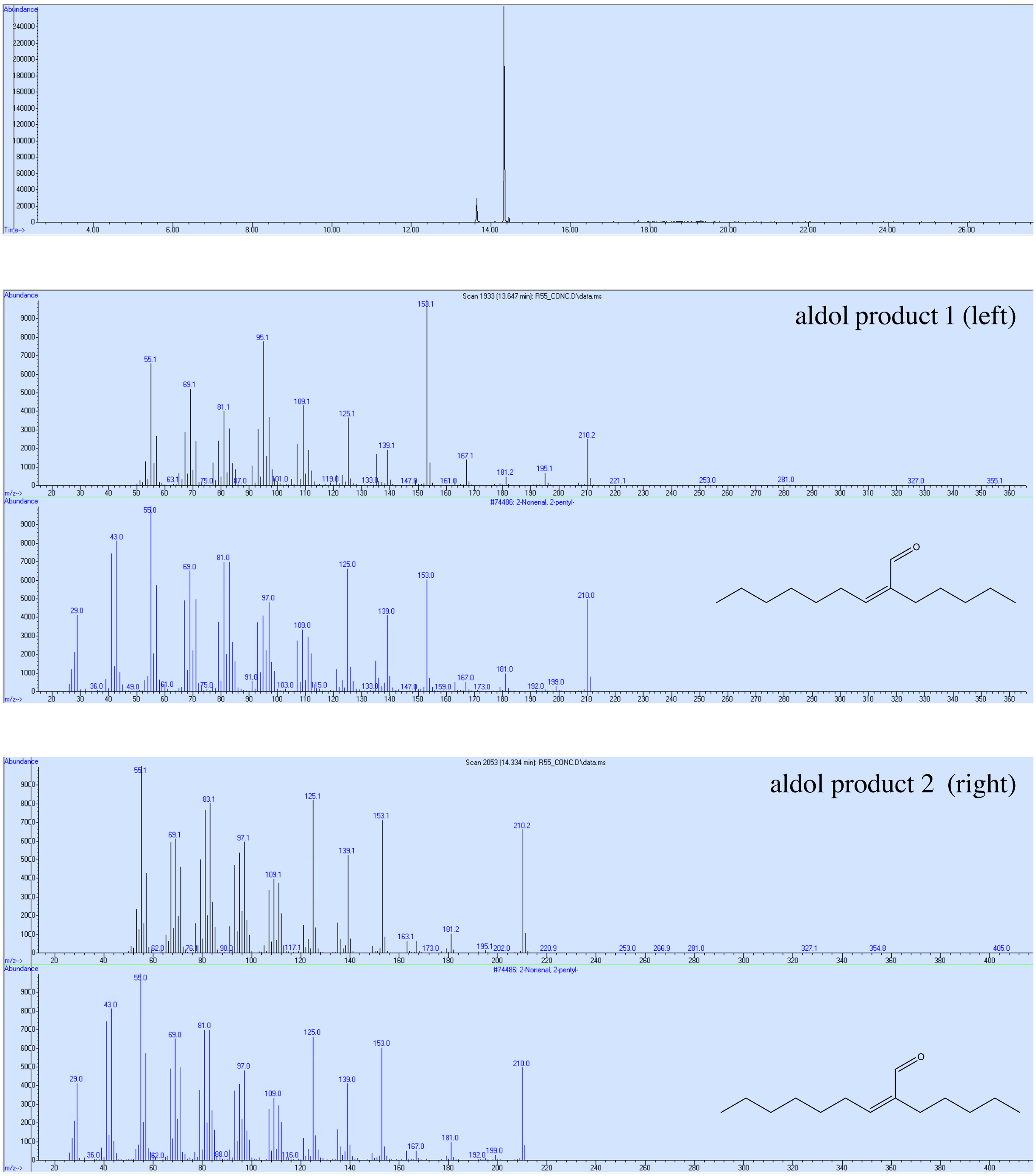

Supplementary Figure 10 Extracted ion chromatogram for the mass of the aldol product (210 u) and mass spectra of the two found compounds compared to the best fitting substance in the database. The two products are likely isomers of the depicted aldol product.

4. Hydroformylation Catalysis Screening

Supplementary Table 5 Screening of reaction conditions in the homogeneous reaction.

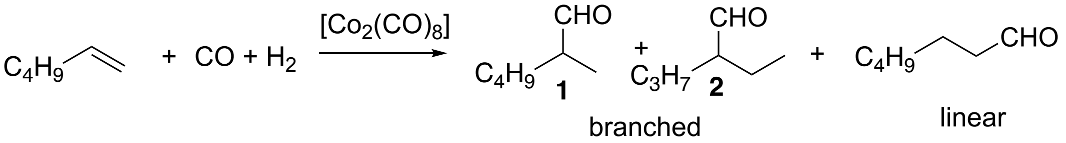

Entry	Co amount (mol %)	Syngas pressure (bar)	Conversion (%)	*B*/*L*
1[a]	0.23	120	n/d	n/d
2[b]	0.23	120	<5%	n/a
3[c],[d]	0.23	19	15	66:34
4[c],[e]	0.23	23	24	45:55
5[c]	0.23	55	62	36:64
6[c]	0.23	94	66	32:68
7[c]	0.06	30	16	49:51
8[c]	0.12	30	35	46:54
9[c]	0.23	30	40	49:51
10[c]	0.47	30	61	54:46
11[c]	1.19	30	>99	54:46
12[c]	2.38	30	>99	51:49

[a] Reaction at 50°C; n/d = not detectable. [b] Reaction at 75°C. [c] Reaction at 100°C. [d].11 bar and [e] 7 bar of Ar were introduced additionally to minimize evaporation of the substrate during the reaction.

Supplementary Table 6 Screening of reaction conditions with MixUMCM-1-NH_2 (28%). Variation of MOF loading and pressure.[a]

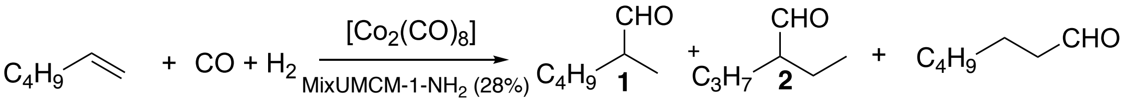

Entry	mol_{MOF}/mol_{Co}	p [bar]	Conversion (%)	*B*/*L*
1	0.8	30	36	75:25
2	1.0	30	28	67:33
3	1.7	30	24	76:24
4	2.8	30	21	72:28
5	1.1	19	10	71:29
6	1.1	30	28	67:33
7	1.1	61	42	51:49
8	1.1	92	61	31:79

[a] $Co_2(CO)_8$ (0.8 mg) was dissolved in 1-hexene (250 µL) and MixUMCM-1-NH_2 (28%) was added; the mixture was brought to various syngas pressure (H_2:CO = 1) at 100°C for 17h

Supplementary Table 7 Blank reaction with different Zn sources.[a]

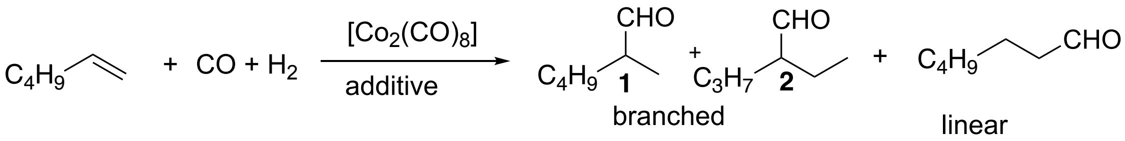

Entry	Additive	mol_{Add}/mol_{Co}	Conversion (%)	*B*/*L*
1	ZnO	5.9	41	53:47
2	$Zn(OAc)_2$*2 H_2O	3.5	9	51:49
3	$Zn(acac)_2$*H_2O	1.8	5	64:36
4	$Zn(NO_3)_2$*6 H_2O	4.6	11	48:52
5	$ZnCl_2$	4.0	11	62:38
6	$ZnSO_4$*7 H_2O	3.0	21	47:53
7	H_3BTB	1.2	35	49:51
8	H_2BDC	2.8	37	54:47
9	H_2BDC-$P(Ph)_2$	1.4	35	49:51

[a] $Co_2(CO)_8$ (0.8 mg) was dissolved in 1-hexene (250 µL) and the additive was added; the mixture was brought to 30 bar syngas (H_2:CO = 1) at 100°C for 17h.

Supplementary Table 8 Blank reaction with different MOFs[a]

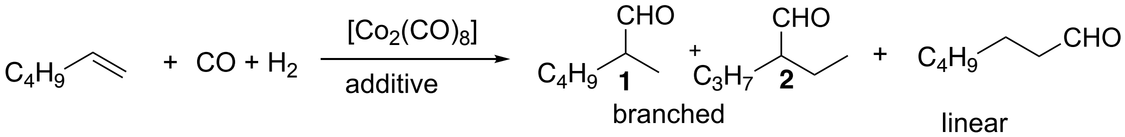

Entry	Additive	mol_{Add}/mol_{Co}	Conversion (%)	*B*/*L*
1	MOF-74(Zn)	41.3	26	85:15
2	UMCM-1	1.7	32	60:40
3	MixUMCM-1-NH_2 (28%)	1.7	24	76:24
4	UMCM-1-NH_2	1.7	20	76:24
5	MOF-74(Mg)	51.7	8	77:23
6	MOF-74(Co)	36.5	90	56:44
7	MOF-74(Ni)	41.3	45	55:45
8[b]	MIL-101(Al)	2.0	<5	n/a
9[b]	MIL-101(Cr)	1.8	<5	n/a
10[b]	Zeolite-Y	n/a	<5	n/a
11	MixUMCM-1-PPh_2 (29 %)	0.9	22	67:33
12	$HCo(CO)_3$(MixUMCM-1-PPh_2)[c]	n/a	9	50:50

[a] $Co_2(CO)_8$ (0.8 mg) was dissolved in 1-hexene (250 μL) and the additive was added; the mixture was brought to 30 bar syngas (H_2:CO = 1) at 100°C for 17h.[b] The reaction was monitored after 17h, 24h, and 48h with no change in results. [c] The material was directly added as catalyst without the addition of $Co_2(CO)_8$

Supplementary Table 9 Reactions at different pressures[a]

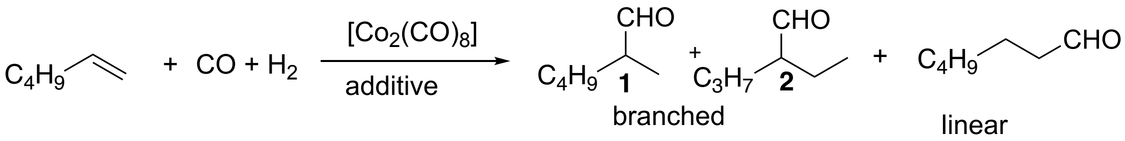

Entry	Additive	Pressure at 100 °C (bar)	Conversion (%)	*B*/*L*
1	/	19	15	66:34
2	/	30	40	49:51
3	/	61	79	34:66
4	/	92	84	27:73
5	MixUMCM-1-NH_2 (28%)[b]	19	10	71:29
6	MixUMCM-1-NH_2 (28%)[b]	30	28	67:33
7	MixUMCM-1-NH_2 (28%)[b]	61	42	51:49
8	MixUMCM-1-NH_2 (28%)[b]	92	61	31:79
9	MOF-74(Zn)	19	12	83:17
10	MOF-74(Zn)	30	25	85:15
11	MOF-74(Zn)	61	55	55:45
12	MOF-74(Zn)	92	62	41:59

[a] $Co_2(CO)_8$ (0.8 mg) was dissolved in 1-hexene (250 μL) and the additive was added; the mixture was brought to the pressure of syngas (H_2:CO = 1) and then heated at 100°C for 17h. [b] mol_{MOF}/mol_{Co} = 1.1. [c] mol_{MOF}/mol_{Co} = 20.

Supplementary Table 10 Aldehyde yields in Table 3 of the main text deterimined by GC-FID with p-cymene as external standard[a]

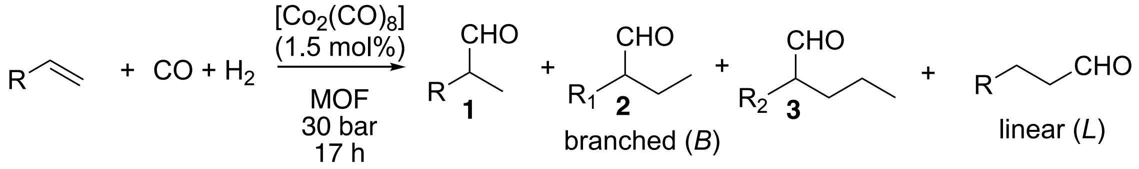

Entry	Olefin	GC aldehydes yield [b]		
		MixUMCM-1-NH$_2$ (28%)[c]	MOF-74(Zn) [d]	No MOF
1	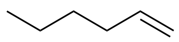	24	17	45
2	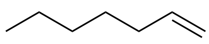	45	24	96
3	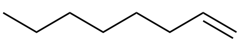	32	25	77
4	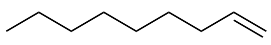	63	47	>99
5	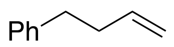	n.a.	n.a.	n.a.

[a] $Co_2(CO)_8$ (1.5 mol%) were dissolved in olefin (500 µL) and the MOF was added. The mixture was brought to 30 bar and then heated to 100°C for 17 h. [b] Determined by GC-FID with calibrated linear aldehyde with *p*-cymene as internal standard. [c] $mol_{MOF}/mol_{Co} = 0.4$. [d] $mol_{MOF}/mol_{Co} = 3.3$.

Supplementary Table 11 Reactions with incipient-wetness impregnated MOFs[a]

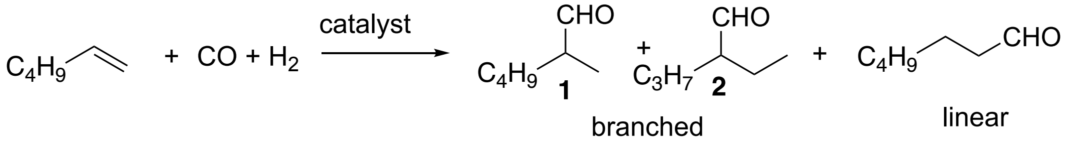

Entry	Catalyst	Co@MOF amount (mg)	Co mol%	Conversion (%)	*B*/*L*
1	Co@MixUMCM-1-NH_2-13	7	0.17	37	61:39
2	Co@MixUMCM-1-NH_2-13	15	0.33	40	65:35
3	Co@MixUMCM-1-NH_2-13	20	0.46	41	68:32
4	Co@MixUMCM-1-NH_2-13	23	0.50	47	68:32
5	Co@MOF-74(Zn)-9.3	20	0.30	50	60:40
6	Co@MOF-74(Zn)-9.3	40	0.60	63	68:32
7	Co@MOF-74(Zn)-4.3	19	0.13	16	51:49
8	Co@MOF-74(Zn)-4.3	41	0.27	20	54:46
9	Co@MOF-74(Zn)-3.8	19	0.11	7	52:48
10	Co@MOF-74(Zn)-3.8	41	0.24	11	53:47

[a] 1-Hexene (500 µL) was added to the catalyst; the mixture was brought to 30 bar pressure of syngas (H_2:CO = 1) at room temperature and then heated at 100°C for 17h.

Supplementary Table 12 Reactions with recycled incipient-wetness impregnated MOFs[a]

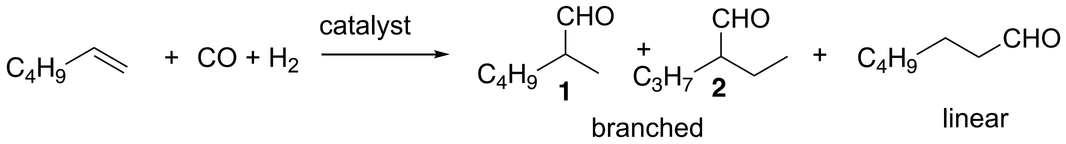

Entry	Catalyst	Co mol% initial	Conversion (%)	*B*/*L*
1	Co@MixUMCM-1-NH_2-13	0.17	traces	~ 50:50
2	Co@MixUMCM-1-NH_2-13	0.33	traces	~ 50:50
3	Co@MixUMCM-1-NH_2-13	0.46	traces	~ 50:50
4	Co@MOF-74(Zn)-4.3	0.27	traces	~ 50:50
5	Co@MOF-74(Zn)-3.8	0.11	traces	~ 50:50
6	Co@MOF-74(Zn)-3.8	0.24	traces	~ 50:50

[a] After the first catalytic run, 1-Hexene (500 μL) was added to the catalyst; the mixture was brought to 30 bar pressure of syngas (H_2:CO = 1) at room temperature and then heated at 100°C for 17h.

Supplementary Table 13 Reactions with recycled, washed MOFs[a]

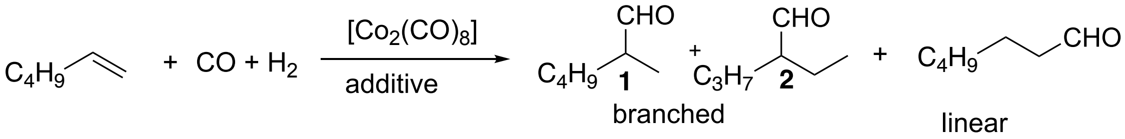

Entry	Additive	Conversion (%)	*B*/*L*
1	MixUMCM-1-NH_2-Recyc	5	64:36
2	MOF-74(Zn)-Recyc	35	68:32

[a] After the first catalytic run, $Co_2(CO)_8$ (0.25 mol%) was dissolved in 1-hexene (500 µL) and the MOF (20 mg) was added. The mixture was brought to 25 bar and then heated to 100°C for 17 h.

5. Computational Details

5.1. Interaction Energy Calculations:

All calculations were performed using the code CP2K [8] at density functional level of theory. The semi-local PBEsol functional was adopted [9] using the DZVP-MOLOPT-SR-GTH gaussian basis set for all the atom types, [10] and a cutoff of 500 Ry for the plane wave auxiliary basis set. The MOFs experimental structures were used as starting point and the catalyst was manually added close to the adsorption/binding site of the MOF. To relieve the computational cost of the calculations, all the systems were studied using the primitive cell. Due to its small size in the a direction, for MOF-74 a supercell 2 x 1 x 1 was adopted. Full geometry optimizations (i.e. both atomic positions and cell parameters) were performed to optimize the MOF-catalyst system. Single point calculations were then performed to calculate the interaction energies between the MOF and the catalyst including the basis-set superimposition error.

In CP2K the interaction energy can be calculated defining 2 fragments A and B. Two fragments corresponding to the MOF (E_A) and the catalyst (E_B) were defined in each case.

$$E_{int} = E_{AB} - (E_A + E_B) \qquad \textbf{(1)}$$

The adsorption/binding sites tested are shown Supplementary Figures 11 to 17 .

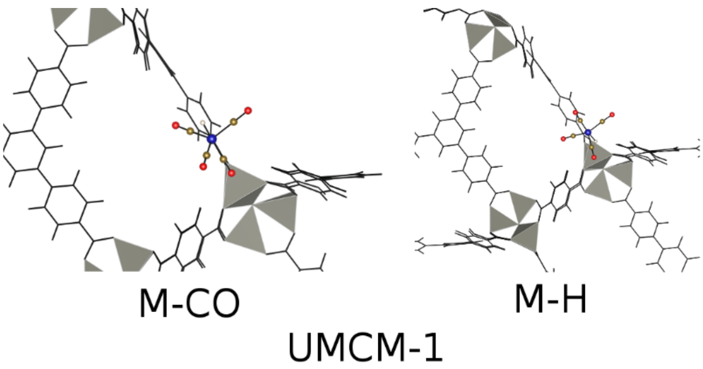

Supplementary Figure 11 Optimised adsorption geometry of $HCoCO_4$ with UMCM-1. Colour scheme: Co = blue, O = red, C = brown, H = white. The organic ligand and inorganic node of the MOF are represented as black line and grey polyhedral for clarity.

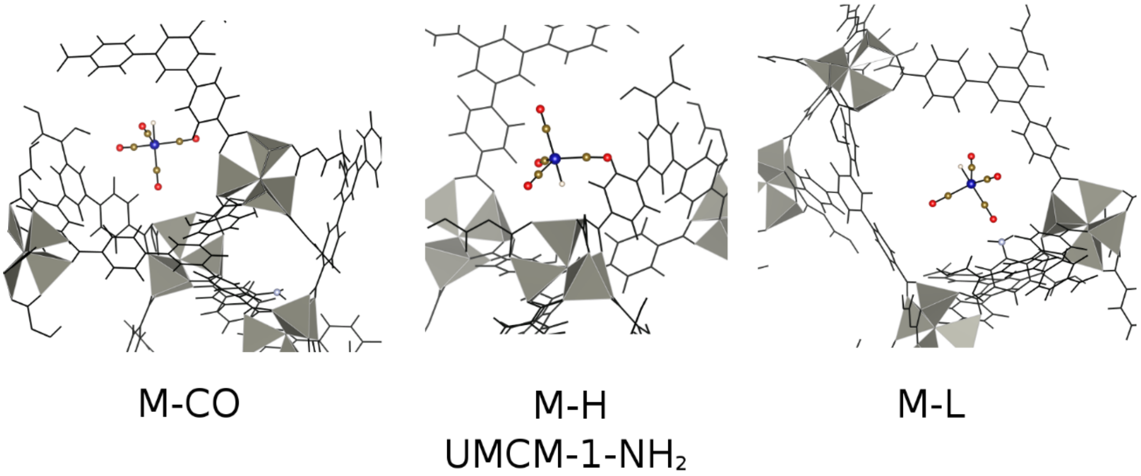

Supplementary Figure 12 Optimised adsorption geometry of $HCoCO_4$ and UMCM-1-NH_2. Colour scheme: Co = blue, O = red, C = brown, H = white. The organic ligand and inorganic node of the MOF are represented as black line and grey polyhedral for clarity.

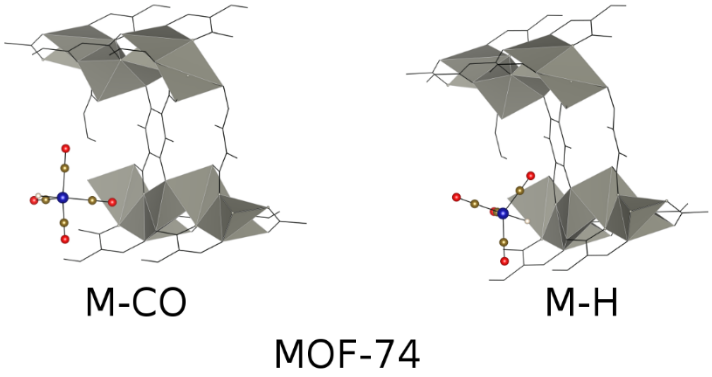

Supplementary Figure 13 Optimised adsorption geometry of $HCoCO_4$ and MOF-74(Zn). Colour scheme, Co = blue, O = red, C = brown, H = beige. The organic ligand and inorganic node of the MOF are represented as black line and grey polyhedral for clarity.

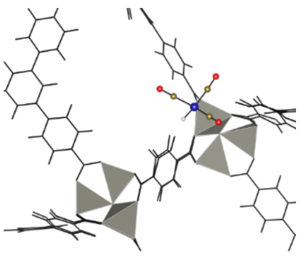

Supplementary Figure 14 Optimised binding geometry of $HCoCO_3$ and UMCM-1. Colour scheme: Co = blue, O = red, C = brown, H = beige. The organic ligand and inorganic node of the MOF are represented as black line and grey polyhedral for clarity.

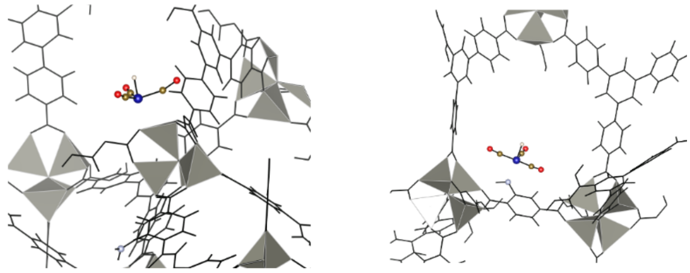

Supplementary Figure 15 Optimised binding geometry of $HCoCO_3$ and UMCM-1-NH_2. Colour scheme: Co = blue, O = red, C = brown, H = beige, N = pale blue. The organic ligand and inorganic node of the MOF are represented as black line and grey polyhedral for clarity.

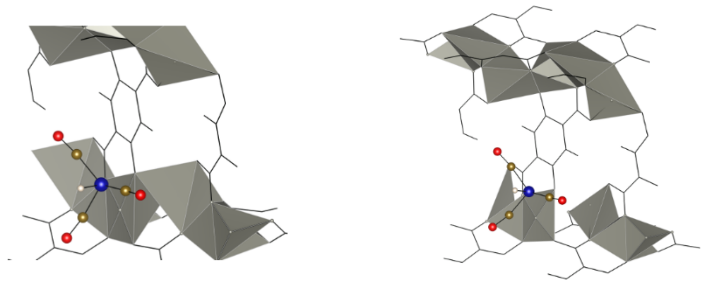

Supplementary Figure 16 Optimized binding geometry of $HCoCO_3$ and MOF-74(Zn). Colour scheme: Co = blue, O = red, C = brown, H = beige. The organic ligand and inorganic node of the MOF are represented as black line and grey polyhedral for clarity.

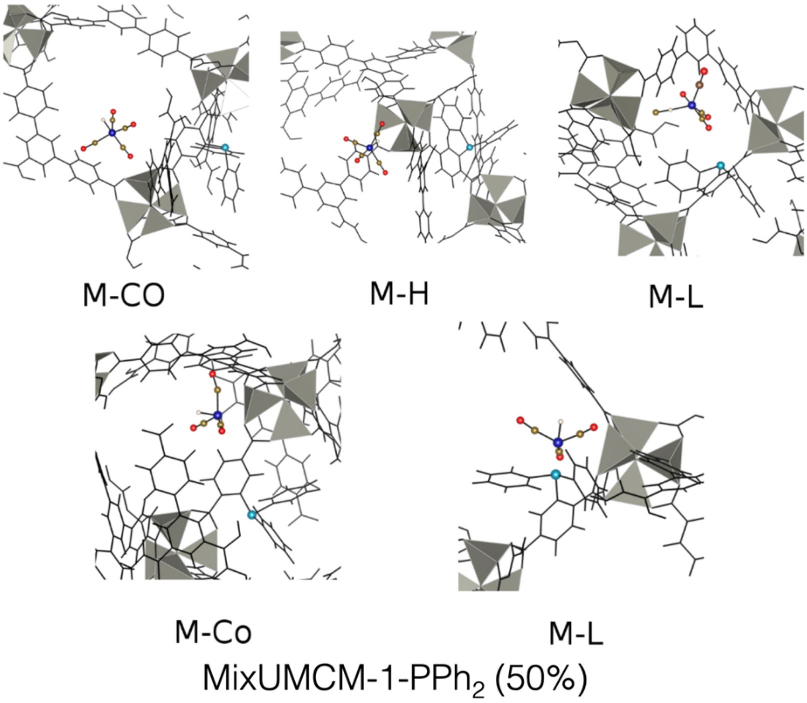

Supplementary Figure 17 On the top, optimized geometry of $HCoCO_4$ and MixUMCM-1-PPh_2 (50%). On the bottom, optimized binding geometry of $HCoCO_3$ and MixUMCM-1-PPh_2 (50%). Colour scheme: Co = blue, O = red, C = brown, H = beige, P = bright blue. The organic ligand and inorganic node of the MOF are represented as black line and grey polyhedral for clarity reason.

5.2. Monte Carlo Simulations

Raspa 2.0 package was used for Monte Carlo simulations.[11] The interactions are computed using Lennard-Jones potential with a cutoff of 14.0 Angstroms for dispersions and a Coulombic potential for charges. As for framework's atom types, the parameters for dispersions are taken from DREIDING force field [12] integrated with UFF[13] for the missing atom types (Mg, Co, Ni and Zn). The choice of the force field is motivated by the good match with adsorption experiments of alkanes in MOF-type frameworks.[14] The point charges are computed using the REPEAT scheme [15] to fit the PBEsol electrostatic potential from geometry optimized structures. These periodic DFT calculations were run using CP2K software package, utilizing the DZVP-MOLOPT-SR-GTH gaussian basis set for all the atom types and a cutoff of 500 Ry for the plane wave auxiliary basis set. The position of the frameworks' atoms is kept fixed in

all the calculation. 1-hexene and aldehydes are modelled using TraPPE force field,[16][17] where the only missing parameters are for the O-CH-CH-CHx torsion potential of the carbonyl group of branched aldehydes. These parameters were computed from quantum mechanics using the MP2/6-31G* method in Gaussian09 (Supplementary Figure 18) coherently with the TraPPE parametrization. Due to the similarity of the two species, only one of the two branched aldehydes is considered: **1** (2-methylhexanal) which is experimentally obtained with the highest ratio.

CO and H_2 molecules are treated as rigid particles. The CO interaction parameters adopted in this work were specifically designed for adsorption in MOF-type materials.[18] The parameters for H_2 are taken from the work of Marx et al.[19] and already validated for adsorption in MOFs.[20][21]

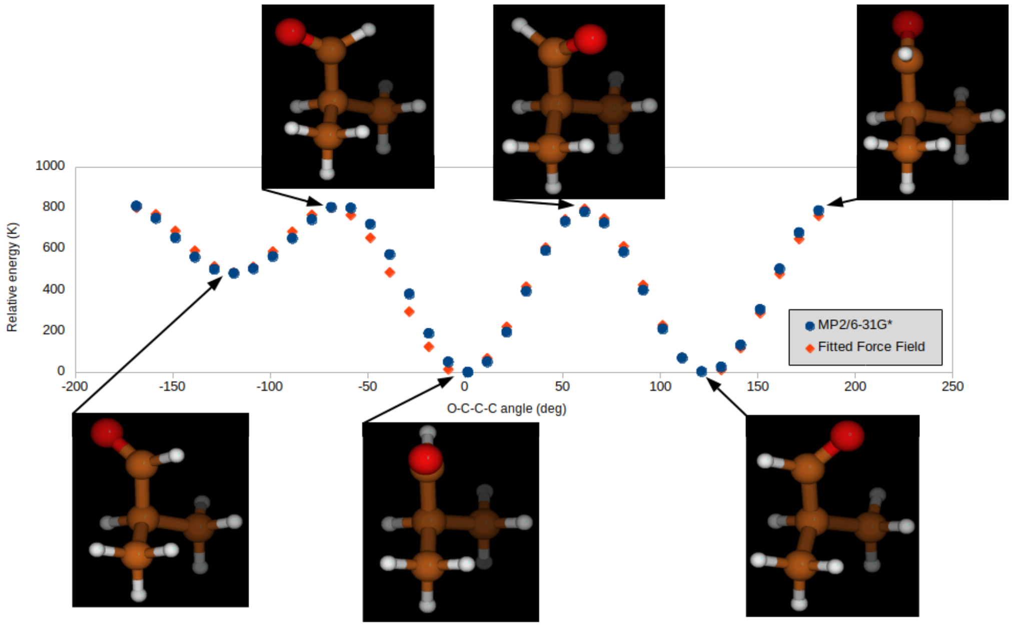

Supplementary Figure 18 Torsional scan of the carbonyl group in a representative model for branched aldehydes. The torsional potential was computed using the MP2/6-31G* method and used to fit the parameters for the TraPPE force field. The plot shows the agreement of the fitting and the conformation of the molecule in the maximum and minimum points

In the following paragraph we describe the protocol used to compute the affinity of the reactive species with the frameworks. First, the amount of 1-hexene inside the bulk frameworks at 30 bar and 100°C was obtained for each considered MOF by performing a Grand Canonical Monte Carlo (GCMC) simulation,[22] where the fugacity of the solvent is derived using the Peng Robinson equation of state.[23] The results of GCMC simulation were averaged for 5,000 cycles, after other 5,000 cycles of initialization. Depending on the concentration of 1-hexene in the framework we computed the volume of the cubic box that simulates the homogenous phase, imposing the same number of molecules and the homogenous density of 1-hexene as computed from GCMC in the empty box (0.00395 molecules per cubic Angstrom). Note that this corresponds to a macroscopic density of 6.56 mol/L, which is slightly smaller than the reported experimental value of 7.11 mol/L. However, to be internally consistent with the simulations we keep the value of 6.56 mol/L as the reference density for the homogeneous phase.

To compute the affinity with the frameworks, one molecule for each reacting component (i.e., H_2, CO, linear heptanal and branched aldehyde **1**) was added to the mixture with saturated 1-hexane. The two simulation boxes, for the homogeneous and the crystal bulk phases, can exchange molecules according to the following rules: 1-hexene and aldehydes were allowed to swap identity between the two boxes and the gas molecules were allowed to be removed and reinserted in the other box. These two moves were possibly selected within the Monte Carlo cycle and attempted, similarly to the other standard molecular moves, i.e., translation, rotation, intramolecular displacement and reinsertion. The choice for the swap rules follows from the consideration that 1-hexene and aldehydes have a similar size and therefore there is a higher probability that a change of identity between them will be accepted, while for the smaller H_2 and CO molecules, it is easier to find interstices between the solvent molecules to be inserted.

For each pair of systems (i.e. MOF + homogenous), we ran ten independent simulations, executing 5,000 cycles of equilibration and 5,000 cycles of production. The final average and standard deviation for the reactant/product occupation is obtained by considering the output these ten runs for the block averaging, i.e., as the result of 50.000 production cycles. Since there are two molecules for each reactive species for each pair of systems (i.e., the framework and the homogenous box) we define as occupancy percentage (%occup.) the averaged probability to find the specie in the MOF's pore volume, divided by two. Therefore, one can observe %occup.=50% if the affinity is the same with both the system (i.e., on average there is one molecule of the specie per box). This is what we obtained when running the simulation for two equivalent boxes, that can be two homogenous boxes or two boxes modelling the bulk of the same MOF. One can observe %occu.→100% when the species has a strong affinity with the MOF (i.e., there is a high probability of finding the two molecules of the species in the MOF's box). Finally, one can observe %occu.→0% when the species has a weak affinity with the MOF, relatively to the homogenous 1-hexane phase (i.e., there is a high probability of finding the two molecules of the species in the homogenous box).

To understand the reason of the higher affinity of the aldehydes with the MOFs, we performed extra calculations on MOF-74(Zn) and UMCM-1, by switching off the coulombic interactions in the simulation. The results reported in Supplementary Table 18, compared with the ones computed from the charged model, show a significant reduction of the MOF affinity with the aldehydes when the carbonyl group is not interacting with the electrostatic potential in the pore volume of the frameworks. According to the TraPPE force field for aldehydes, the CH bead and the oxygen have a charge of 0.525 and -0.482, respectively. The Zn charge computed using the REPEAT method is 1.190 in MOF-74 and 1.27 in UMCM-1.

The MOF-aldehyde affinity is therefore enthalpic and apparently not dependent on the type of isomer (linear or branched), i.e., it is not due to a steric confinement as in the case of "shape selectivity" seen for hydrocarbons in zeolites. In the non-charged system, the affinity of the gas molecule that are only weakly polar, remains almost unvaried and suggests that there is a minor effect due to non-covalent interactions. We conclude that the higher affinity of the gas molecules with the liquid 1-hexene is due to an entropic motivation. The 1-hexene saturated in the pore volume has a higher density and the confinement effect is higher inside the MOF: this results in a lower probability of forming interstices where the small gas molecules can fit, i.e., the cavitation contribute to the solvation energy of H_2 and CO.

5.3. Pore Volume Calculations

The pore volume in the bulk frameworks, that was used to calculate the density of the saturated 1-hexene is computed with the "probe occupiable pore volume" (VOLPO) routine[24] as implemented in the Zeo++ v0.3 software package.[25][26] For the probing of the volume, 500.000 samples were used, together with the high accuracy (-ha) option in Zeo++. The radii of the atoms in the framework were taken as half of the Lennard-Jones sigma parameter of the force field (DREIDING, integrated with UFF for the missing atom types). This choice is coherent with the potential used for the Monte Carlo simulations.

As for the probe size, a diameter of 3.703 Å was utilized. This value is the average of the sigma parameters for the CH_3 (3.75 Å) and the CH_2 (3.675 Å) beads in TraPPE force field, that are, respectively, the head and the tail of the 1-hexene molecule.

6. DFT Calculations

Supplementary Table 14 DFT interaction energy of $HCo(CO)_4$. M-Co (**MOF–CO-**Co(H)$(CO)_3$ in the main text) is the adsorption of $HCoCO_4$ to the metal node via its axial carbonyl. M-H (**MOF–H**-Co$(CO)_4$ in the main text) is the adsorption of $HCoCO_4$ to the metal node via its hydride. L-CO = adsorption of $HCoCO_4$ to the function via its axial carbonyl. Optimised geometries reported in supplementary notes (Supplementary Figures 11 to 17).

	M-CO (kcal/mol)	M-H (kcal/mol)	L-CO (kcal/mol)
UMCM-1	1.20	0.19	/
UMCM-1-NH_2	0.69	0.01	-0.27
UMCM-1-PPh_2	-0.03	-0.09	-0.09
MOF-74(Zn)	-1.68	-2.53	/

Supplementary Table 15 DFT binding energies. M-Co (**MOF–Co**(H)(CO)$_3$ in the main text) is the binding energy between the metal of the MOF and the Co of $HCoCO_3$. L-Co (MOF**Func–Co**(H)(CO)$_3$ in the main text) is the binding energy between the functional group of the MOF and the Co of $HCoCO_3$. Optimised geometries reported in supplementary notes (Supplementary Figures 11 to 17).

	M-Co	L-Co
UMCM-1	-3.49	/
UMCM-1-NH_2	-20.22	-34.02
UMCM-1-PPh_2	-54.95	-53.39
MOF-74(Zn)	-28.74	-33.84

7. Monte Carlo Simulations

Supplementary Table 16 Grand Canonical Monte Carlo (GCMC) simulations. For each framework the first column lists the 1-hexene uptake measured from Grand Canonical Monte Carlo (GCMC) simulations (average and standard deviation over 5 blocks). The second column shows the rounded number of 1-hexene molecules that were used for the following simulations and to compute the size of the paired homogenous box that contains the same number of solvent molecules, reported in the third column.

Framework	1-hex. uptake (molec./UC)	1-hex. uptake (molec./box)	Homogeneous box volume (Å^3)
UMCM-1	176.0 ± 2.4	176	44.557
UMCM-1-NH_2	176.2 ± 2.4	176	44.557
UMCM-1-PPh_2	161.3 ± 3.5	161	40.759
MOF-74(Zn)	246.8 ± 3.0	247	62.532

Supplementary Table 17 Affinity of the different species with the frameworks. This is reported as percentage occupancy (%occup.) which is related to the average number of molecules of that species in the MOF's simulation box. The error is computed as standard deviation over ten independent simulations. The last column reports the relative density of 1-hexene computed in the pore volume with respect to the density observed in the homogeneous simulation box (see also Supplementary Table 16).

MOF	1-hexene Rel. density	H_2 %occup.	CO %occup.	n-Heptanal %occup.	**1** %occup.
UMCM-1	1.04 ± 0.01	40.0 ± 0.5%	41.3 ± 0.5%	66.7 ± 1.5%	64.0 ± 1.4%
UMCM-1-NH_2	1.04 ± 0.01	39.1 ± 0.1%	40.8 ± 0.5%	67.2 ± 0.7%	64.4 ± 2.2%
UMCM-1-PPh_2	1.04 ± 0.02	40.8 ± 0.5%	41.2 ± 0.6%	68.9 ± 2.2%	66.6 ± 1.9%
MOF-74 (Zn)	1.14 ± 0.01	22.4 ± 0.7%	21.9 ± 1.4%	80.9 ± 1.9%	81.5 ± 0.6%

Supplementary Table 18 The affinity of the different species with UMCM-1 and MOF-74(Zn). as setting to zero the Coulomb interactions. is reported with its standard deviation. The difference in the %occup. with the charged model shown in Supplementary Table 19 is also reported

Species	UMCM-1 (no charges) %occup.	difference	Zn-MOF-74 (no charges) %occup.	difference
Heptanal	55.1 ± 0.4%	-11.6%	66.3 ± 1.5%	-14.3%
2-metylhexanal (**1**)	55.5 ± 1.8%	-8.5%	66.0 ± 1.0%	-15.6%
H_2	40.6 ± 0.2%	+0.6%	20.1 ± 0.7%	-2.3%
CO	41.6 ± 0.7%	+0.3%	20.0 ± 1.3%	-1.9%

8. Kinetic Analysis

The kinetic analysis was based on the empirical rate of formations of the branched and the linear aldehydes reported in the paper.[27] The two equations are shown below and are also reported in the full text.

$$R_B = \frac{k_B \cdot [H_2]^{0.32} \cdot [CO] \cdot [Co_2(CO)_8]^{0.62} \cdot [alkene]}{(1+K_{BCO} \cdot [CO])^2} \quad (2)$$

$$R_L = \frac{k_L \cdot [H_2]^{0.55} \cdot [CO] \cdot [Co_2(CO)_8]^{0.75} \cdot [alkene]^{0.87}}{(1+K_{LCO} \cdot [CO])^2} \quad (3)$$

The kinetic and equilibrium constants at 110 °C were all taken from the publication:

k_B (110 °C) = $2.12 \cdot 10^{-7}(m^3 \cdot mol^{-1}) \cdot 1.94 s^{-1}$

K_{BCO} (110 °C) = $1.35 \cdot 10^{-3} \cdot m^3 \cdot mol^{-1}$

k_L (110 °C) =2 .01·$10^{-7}(m^3 \cdot mol^{-1}) \cdot 2.17 s^{-1}$

K_{LCO} (110 °C) = $8.014 \cdot 10^{-3} \cdot m^3 \cdot mol^{-1}$

The concentration of pure 1-hexene is $7.11 \cdot 10^3$ mol/m^3 at 30 bar and 100 °C (http://www.ddbst.com/en/EED/PCP/DEN_C100.php).

The concentration of H_2 and CO at different pressures in 1-hexene were calculated using the Soave modifications of the Redlich-Kwong equation (SRK)[28] and are reported in Supplementary Table 19.

The concentrations of 1-hexene, CO and H_2 within the pores of the MOFs were calculated by multiplying the concentration in the homogeneous phase by a factor Z derived from the Monte Carlo simulations (Supplementary Table 20). The Z factor for H_2 and CO were calculated by using equation (4). This is consistent with the fact that an %occup. of 50 would give a Z factor of 1 and therefore no preference of a molecule to be either in the homogeneous or the MOF phase, while with %occup. of 0 one would find null concentration inside the MOF as both CO and H_2 are found with %occup. < 50%.

$$Z = \%occup. / 50 \qquad (4)$$

The concentration of $Co_2(CO)_8$ was calculated from the catalyst loading relative to the 1-hexene concentration.

Supplementary Table 19 Molar solubilities of H_2 and CO in 1-hexene at 100 °C in function of pressure.

Pressure (bar)	Molar solubility of H_2 (mol/L)	Molar solubility of CO (mol/L)
10	0.0565855989	0.104422395
20	0.135717257	0.251731862
30	0.214408549	0.399737285
40	0.292661936	0.548459301
50	0.37048037	0.697919993
60	0.447866674	0.848143
70	0.524823672	0.999153594
80	0.60135418	1.15097877
90	0.677463539	1.30365115
100	0.753150085	1.45719046

Supplementary Table 20 Correction factors Z used to calculate the modified concentration of the reactants within the pores of the MOFs.

MOF	Z[1-hexene]	Z[H_2]	Z[CO]
UMCM-1	1.04	0.80	0.82
UMCM-1-NH_2	1.04	0.78	0.82
MixUMCM-1-PPh_2 (50%)	1.04	0.82	0.82
MOF-74(Zn)	1.14	0.45	0.44

8.1. Rates of formation in homogeneous catalysis

[1-hexene] = $7.11 \cdot 10^3$ mol/m^3

[Co_2CO_8] = 8.53 mol/m^3

Supplementary Table 21 Concentration of H_2 and CO at different syngas pressures. (H_2:CO = 1) Their effect on the rate of formations of the branched aldehyde R_B and of the linear one R_L for homogeneous catalysis is shown.

**P Syngas (bar)**	**[H_2] (mol·m^{-3})**	**[CO] (mol·m^{-3})**	***R_B* (mol·m^{-3}·s^{-1})**	***R_L* (mol·m^{-3}·s^{-1})**	***R_B/R_L***
**10**	20.5	24.7	0.208	0.204	1.019
**15**	39.8	62.3	0.340	0.473	0.719
**20**	59.2	99.9	0.380	0.654	0.581
**25**	78.5	137.4	0.387	0.772	0.502
**30**	97.9	175.0	0.382	0.849	0.450
**35**	117.2	212.6	0.371	0.899	0.413
**40**	136.6	250.1	0.358	0.932	0.385
**45**	155.9	287.7	0.345	0.952	0.363
**50**	175.3	325.3	0.333	0.964	0.345
**55**	194.6	362.9	0.320	0.971	0.330
**60**	214.0	400.4	0.309	0.973	0.318
**65**	233.3	438.0	0.298	0.972	0.307
**70**	252.7	475.6	0.289	0.969	0.298
**75**	272.0	513.1	0.279	0.964	0.290
**80**	291.4	550.7	0.271	0.958	0.282
**85**	310.7	588.3	0.263	0.952	0.276
**90**	330.1	625.9	0.255	0.945	0.270
**95**	349.4	663.4	0.248	0.937	0.265
**100**	368.8	701.0	0.241	0.929	0.260

8.2. Rates of formation within the pores of UMCM-1-NH_2

[1-hexene] = $7.39 \cdot 10^3$ mol/m^3

[Co_2CO_8] = 8.87 mol/m^3

Supplementary Table 22 Concentration of H_2 and CO at different syngas pressures. (H_2:CO = 1) Their effect on the rate of formations of the branched aldehyde R_B and of the linear one R_L for catalysis within the pores of UMCM-1-NH_2 is also shown.

**P Syngas (bar)**	**[H_2] (mol·m^{-3})**	**[CO] (mol·m^{-3})**	**R_B (mol·m^{-3}·s^{-1})**	**R_L (mol·m^{-3}·s^{-1})**	**R_B/R_L**	**$\frac{R_B/R_L[MOF]}{R_B/R_L[Homogeneous]}$**
**10**	16.0	20.3	0.184	0.165	1.113	1.093
**15**	31.1	51.1	0.326	0.408	0.798	1.111
**20**	46.2	81.9	0.382	0.589	0.648	1.115
**25**	61.3	112.7	0.401	0.717	0.559	1.114
**30**	76.3	143.5	0.404	0.808	0.500	1.111
**35**	91.4	174.3	0.399	0.872	0.457	1.108
**40**	107.0	205.1	0.390	0.917	0.425	1.106
**45**	122.0	235.9	0.379	0.949	0.400	1.103
**50**	137.0	266.7	0.369	0.971	0.379	1.100
**55**	152.0	297.5	0.358	0.986	0.362	1.098
**60**	167.0	328.4	0.347	0.996	0.348	1.096
**65**	182.0	359.2	0.337	1.002	0.336	1.094
**70**	197.0	390.0	0.327	1.004	0.325	1.092
**75**	212.0	420.8	0.317	1.005	0.316	1.090
**80**	227.0	451.6	0.309	1.003	0.308	1.089
**85**	242.0	482.4	0.300	1.001	0.300	1.088
**90**	257.0	513.2	0.292	0.997	0.293	1.086
**95**	273.0	544.0	0.285	0.992	0.287	1.085
**100**	288.0	574.8	0.278	0.987	0.282	1.084

8.3. Rates of formation within the pores of MOF-74(Zn)

[1-hexene] = $8.10 \cdot 10^3$ mol/m^3

[Co_2CO_8] = 9.72 mol/m^3

Supplementary Table 23 Concentration of H_2 and CO at different syngas pressures. (H_2:CO = 1) Their effect on the rate of formations of the branched aldehyde R_B and of the linear one R_L for catalysis within the pores of MOF-74(Zn) is also shown.

**P Syngas (bar)**	**[H_2] (mol·m^{-3})**	**[CO] (mol·m^{-3})**	***R_B* (mol·m^{-3}·s^{-1})**	***R_L* (mol·m^{-3}·s^{-1})**	***R_B/R_L***	***R_B/R_L[MOF] / R_B/R_L[Homogeneous]***
**10**	9.2	10.9	0.118	0.087	1.363	1.338
**15**	17.9	27.4	0.259	0.251	1.032	1.436
**20**	26.6	43.9	0.348	0.407	0.856	1.472
**25**	35.3	60.5	0.403	0.542	0.744	1.482
**30**	44.0	77.0	0.437	0.657	0.666	1.480
**35**	52.8	93.5	0.457	0.753	0.607	1.472
**40**	61.5	110.1	0.468	0.833	0.562	1.462
**45**	70.2	126.6	0.473	0.899	0.526	1.451
**50**	78.9	143.1	0.474	0.954	0.496	1.439
**55**	87.6	159.7	0.472	1.000	0.472	1.429
**60**	96.3	176.2	0.468	1.039	0.451	1.418
**65**	105.0	192.7	0.463	1.071	0.433	1.409
**70**	114.0	209.3	0.458	1.097	0.417	1.400
**75**	122.0	225.8	0.451	1.120	0.403	1.392
**80**	131.0	242.3	0.445	1.138	0.391	1.384
**85**	140.0	258.8	0.438	1.153	0.380	1.377
**90**	149.0	275.4	0.431	1.166	0.370	1.370
**95**	157.0	291.9	0.424	1.176	0.361	1.364
**100**	166.0	308.4	0.418	1.184	0.353	1.358

9. Supplementary References

[1] K. Ito. N. Kamiyama, S. Nakanishi, Y. Otsuji.,*Chem. Lett.* **1983**, 657-660.

[2] M. Servalli, M. Ranocchiari, J. A. Van Bokhoven, *Chem. Comm.* **2012**, *48*, 1904-1906.

[3] F.L. Morel, M. Ranocchiari, J.A. van Bokhoven, *Ind. End. Chem. Res.* **2014**, *53*, 9120-9127.

[4] G. Bauer, D. Ongari, X. Xu, D. Tiana, B. Smit, M. Ranocchiari, *J. Am. Chem. Soc.* **2017**, *139*, 18166-18169.

[5] P. Serra-Crespo, E.V. Ramos-Fernandez, J. Gascon, F. Kapteijn, *Chem. Mater.*, **2011**, *23*, 2565–2572.

[6] S. Sorribas, P. Gorgojo, C. Téllez, J. Coronas, A.G. Livingston, *J. Am. Chem. Soc.*, **2013**, *135*, 15201–15208.

[7] X. Wu, Z. Bao, B. Yuan, J. Wang, Y. Sun, H. Luo, S. Deng, *Microporous Mesoporous Mater.*, **2013**, *180*, 114–122.

[8] J. Hutter, M. Iannuzzi, F. Schiffmann, J. Vandevondele *Wires Comput. Mol. Sci.*, **2014**, *4*, 15–25

[9] J.P. Perdew, A. Ruzsinszky, G.I. Csonka, O.A. Vydrov, G.E. Scuseria, L.A. Constantin, X. Zhou, K. Burke *Phys. Rev. Lett.*, **2007**, *100*, 136406.

[10] J. Vandevondele, J. Hutter *J. Chem. Phys.*, **2017**, *127*, 114105-114109.

[11] D. Dubbeldam, S. Calero, D.E. Ellis, R.Q. Snurr *Molec. Simul.*, **2016**, *42*, 81-101.

[12] S.L. Mayo, B.D. Olafson, W.A. Goddard *J. Phys. Chem.*, **1990**, *94*, 8897–8909.

[13] A.K. Rappe, C.J. Casewit, K.S. Colwell, W.A. Goddard Iii, W.M. Skiff *J. Am. Chem. Soc.*, **1992**, *114*, 10024–10035.

[14] I. Déroche, S. Rives, T. Trung, Q. Yang, A. Ghoufi, N. A. Ramsahye, P. Trens, F. Fajula, T. Devic, C. Serre, G. Férey, H. Jobic, And G. Maurin *J. Phys. Chem. C*, **2011**, *115*, 13868–13876.

[15] C. Campañá, B. Mussard, T.K. Woo *J. Chem. Theory Comput.*, **2009**, *5*, 2866–2878.

[16] C.D. Wick, M.G. Martin, J.I. Siepmann, *J. Phys. Chem. B*, **2000**, *104*, 8008–8016.

[17] J.M. Stubbs, J.J. Potoff, J.I. Siepmann, *J. Phys. Chem. B*, **2004**, *108*, 17596–17605.

[18] A. Martín-Calvo, F.D. Lahoz-Martín, S. Calero, *J. Phys. Chem. C*, **2012**, *116*, 6655–6663.

[19] D. Marx, P. Nielaba, *Phys. Rev. A*, **1992**, *45*, 8968-8961.

[20] N. Bobbitt, J. Chen, R. Snurr, *J. Phys. Chem. C,* **2006**, *120*, 27328-27341.

[21] B.J. Bucior, N. S. Bobbitt, T. Islamoglu, S. Goswami, A. Gopalan, T. Yildirim, O.K. Farha, N. Bagheri, R.Q. Snurr, Mol. Syst. Des. Eng., **2019**, 4, 162-174.

[22] D. Frenkel, B.Smit. (2002). *Understanding molecular simulation : From algorithms to applications* (2nd ed., Vol. Vol. 1, Computational science). San Diego: Academic Press.

[23] D.Y. Peng, D.B. Robinson, *Ind. Eng. Chem. Fundamen.*, **1976**, *15*, 59–64.

[24] D. Ongari, P.G. Boyd, S. Barthel, M. Witman, M. Haranczyk, B. Smit, *Langmuir*, **2017**, *33*, 14529–14538.

[25] T.F. Willems, C.H. Rycroft, M. Kazia, J.C. Meza, M. Haranczyk, *Microporous Mesoporous Mater.*, **,** 149, 134-141.

[26] http://www.zeoplusplus.org/ Last accessed at 13.11.2018.

[27] R. V. Gholap, O. M. Kut, , J. R. Bourne, *Ind. Eng. Chem. Res.* **1992**, 31 (11), 2446–2450.

[28] P. M. Mathias, T. W. Copeman *Fluid Phase Equilibria* **1983**, 13, 91–108.
